# Effects of ulotaront on brain circuits of reward, working memory, and emotion processing in healthy volunteers with high or low schizotypy

**DOI:** 10.1038/s41537-023-00385-6

**Published:** 2023-08-07

**Authors:** Francesca Perini, Jadwiga Maria Nazimek, Shane Mckie, Liliana P. Capitão, Jessica Scaife, Deepa Pal, Michael Browning, Gerard R. Dawson, Hiroyuki Nishikawa, Una Campbell, Seth C. Hopkins, Antony Loebel, Rebecca Elliott, Catherine J. Harmer, Bill Deakin, Kenneth S. Koblan

**Affiliations:** 1 Faculty of Biology, Medicine and Health, Division of Neuroscience and Experimental Psychology, School of Biological Sciences, University of Manchester, Manchester Academic Health Science Centre, Manchester, M13 9PT UK; 2 University Department of Psychiatry, University of Oxford and Oxford Health NHS Foundation Trust, Warneford Hospital, Warneford Lane, Oxford, OX3 7JX UK; 3 P1vital LTD, Manor House, Howbery Business Park, Wallingford, OX10 8BA UK; 4 Sunovion Pharmaceuticals Inc., 84 Waterford Drive, Marlborough, MA 01752 USA

**Keywords:** Schizophrenia, Pharmacology

## Abstract

Ulotaront, a trace amine-associated receptor 1 (TAAR1) and serotonin 5-HT1A receptor agonist without antagonist activity at dopamine D_2_ or the serotonin 5-HT2A receptors, has demonstrated efficacy in the treatment of schizophrenia. Here we report the phase 1 translational studies that profiled the effect of ulotaront on brain responses to reward, working memory, and resting state connectivity (RSC) in individuals with low or high schizotypy (LS or HS). Participants were randomized to placebo (*n* = 32), ulotaront (50 mg; *n* = 30), or the D_2_ receptor antagonist amisulpride (400 mg; *n* = 34) 2 h prior to functional magnetic resonance imaging (fMRI) of blood oxygen level-dependent (BOLD) responses to task performance. Ulotaront increased subjective drowsiness, but reaction times were impaired by less than 10% and did not correlate with BOLD responses. In the Monetary Incentive Delay task (reward processing), ulotaront significantly modulated striatal responses to incentive cues, induced medial orbitofrontal responses, and prevented insula activation seen in HS subjects. In the N-Back working memory task, ulotaront modulated BOLD signals in brain regions associated with cognitive impairment in schizophrenia. Ulotaront did not show antidepressant-like biases in an emotion processing task. HS had significantly reduced connectivity in default, salience, and executive networks compared to LS participants and both drugs reduced this difference. Although performance impairment may have weakened or contributed to the fMRI findings, the profile of ulotaront on BOLD activations elicited by reward, memory, and resting state is compatible with an indirect modulation of dopaminergic function as indicated by preclinical studies. This phase 1 study supported the subsequent clinical proof of concept trial in people with schizophrenia.

**Clinical trial registration:** Registry# and URL: ClinicalTrials.gov NCT01972711, https://clinicaltrials.gov/ct2/show/NCT01972711

## Introduction

Ulotaront is a trace amine-associated receptor 1 (TAAR1) agonist with serotonin 5-HT1A agonist activity, but without affinity for D_2_ or serotonin 5-HT2A receptors, the targets of current antipsychotics^1^. TAARs are a family of G-protein-coupled receptors; the TAAR1 subtype, expressed in multiple brain regions including key dopaminergic nuclei (ventral tegmental area [VTA]) and serotonergic (dorsal raphe nuclei [DRN]), modulates monoaminergic and glutamatergic neurotransmission^2^. In mice, ulotaront exerts inhibitory effects on VTA neuronal firing^1^ and attenuates the ketamine-induced increase in striatal dopamine synthesis capacity^3^. In addition, ulotaront prominently suppresses rapid eye movement (REM) sleep in rodents and humans, similar to many antidepressants^4^. Ulotaront has demonstrated broad efficacy in preclinical models of psychosis^1^ and is currently in phase 3 clinical trials evaluating its safety and efficacy in the treatment of schizophrenia, with positive results available from both an initial acute treatment study^5^ and a 6-month continuation study^6^. Here we review the phase 1 studies that led to phase 2 clinical development.

Schizotypy is a dimensional personality trait that shares genetic, neuroanatomical, neurobiological, and cognitive characteristics with schizophrenia and schizophrenia spectrum disorders^7–9^. Subjects with personality trait scores demonstrating high schizotypy (HS) versus low schizotypy (LS) were recruited to widen the magnitude of the changes in reward processing and executive function analogous to those seen in schizophrenia, but in people who are entirely healthy and without the confounds of drug treatment and psychotic symptoms.

In light of the preclinical results, we undertook phase I translational studies, using functional magnetic resonance imaging (fMRI) in healthy volunteers with high or low schizotypy, that were designed to evaluate the effects of ulotaront compared to the D_2_ receptor antagonist amisulpride^10^ on neurocognitive processes implicated in the pathogenesis of schizophrenia, including brain circuits of reward, working memory and emotional processing, resting state connectivity (RSC), and cerebral blood flow (CBF).

The primary outcome was blood oxygen level‒dependent (BOLD) responses in ventral striatum (VS) during the anticipation phase of the monetary incentive delay (MID) task. This task has been widely used in fMRI studies to investigate reward circuitry in response to anticipation of wins or losses and their occurrence^11–13^. Cues that predict monetary wins elicit increased BOLD responses in the VS that are mediated by increased dopamine release and thus attenuated by postsynaptic dopamine receptor-blocking antipsychotics. It is thought that high-tonic dopamine release obscures contingent release and results in random learning of incentive salience and thus the positive symptoms of schizophrenia^14^. Increased VS activity appears to be a shared neuronal correlate of positive symptoms in schizotypal people and unmedicated first episode patients^15^. We hypothesized that ulotaront would attenuate VS BOLD responses in the anticipatory phase of the MID task in view of its preclinical actions in decreasing dopamine neuronal firing and synthesis capacity^1,3^.

Secondary outcomes included ulotaront and amisulpride effects on fMRI responses during N-Back working memory task and neurocognitive processes of identification and recall of emotionally-valenced faces or words in the emotional test battery (ETB). The N-Back signal detection task has been used to assess impairments of working memory, associated with poor social and occupational functioning in schizophrenia^16^. We previously showed that amisulpride (400 mg) reversed impaired performance in high schizotypes performing this task^17^ and hypothesized ulotaront would have the same effect. We used the ETB to assess possible antidepressant efficacy given the antidepressant-like preclinical findings. Using the ETB, acute or 7-day administration of antidepressants, SSRIs or SNRIs, was shown to increase the recall of positive self-referent words and the perception of ambiguous faces as happy^18–21^ in healthy volunteers and patients with major depressive disorders.

Finally, we conducted exploratory analyses of RSC and arterial spin labeling. There is evidence of abnormal RSC^22,23^ within the default mode network (DMN, self-referential processing), executive control network (ECN, goal-directed activity) and anterior salience network (ASN, detecting and orienting towards salient stimuli)^24–26^ in schizophrenia. In addition, arterial spin labeling (ASL) provides absolute measures of CBF, reflecting regional neuronal activity. A recent study found a specific association between increased CBF in striatum and the negative symptom, apathy^27^. Another study found alterations in striatal and prefrontal CBF may precede onset of psychosis^28^.

## Materials and methods

### Participants

**Fig. 1:**
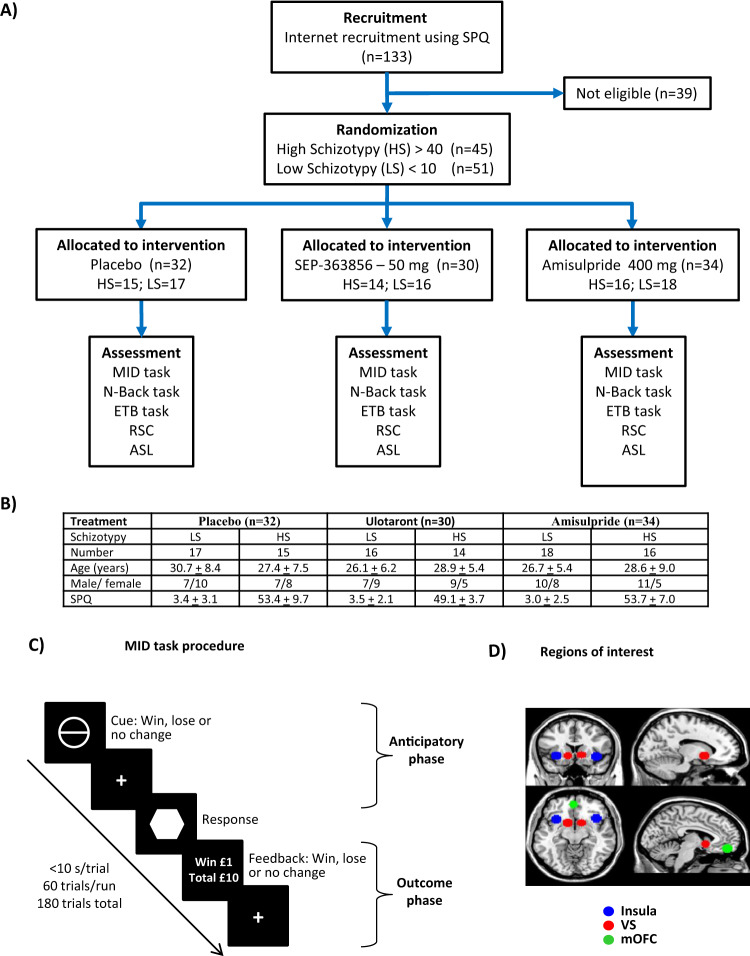
Study design and task procedures. **A** Experimental workflow diagram showing screening for schizotypy and allocation to treatment groups for selected volunteers. **B** Demographic data, including treatment group, age, gender, and level of schizotypy in the MID task. **C** Design of the MID task. Participants were presented with a cue predicting either monetary gain, loss, or no change, and asked to press a button in response to a following target as fast as possible. There were three types of trial: possible reward, possible punishment and neutral, depending on whether participants responded on time to the target. **D** ROI used in the MID task. Masks were created in bilateral striatum, bilateral insula and mOFC. All masks were 10-mm radius spheres based on previously reported coordinates from the meta-analysis of Liu et al.^41^. Specific coordinates of ROI used for the anticipatory phase were 38 20 –8, and -32 18 –6 for right and left insula, 12 10 –4 and –12 10 –6 for right and left striatum, and 2 50 –16 for mOFC. For the outcome phase, coordinates were 36 22 –8 and –28 24 –8 for right and left insula, 12 10 –6 and 10 8 –4 for right and left striatum and –2 56 –6 and 2 48 –14 for mOFC. ASL arterial spin labeling, ETB emotional test battery, MID monetary incentive delay, mOFC medial orbital frontal cortex, RSC resting state connectivity, SPQ Schizotypal Personality Questionnaire, VS ventral striatum.

The study was designed and conducted in accordance with the principles of the Declaration of Helsinki (1964) and registered with ClinicalTrials.gov, identifier: NCT01972711. This was a double-blind, placebo-controlled, randomized study performed at the Universities of Manchester and Oxford, UK between March 27, 2014 and July 7, 2015. The study was approved by the National Research Service (Northwest). Three amendments to the protocol were approved to clarify the population to be studied, amend timing of the interim analysis, and permit greater flexibility in scheduling. All participants gave informed consent. Baseline and final visit health checks included physical and neurological examinations; electrocardiography; renal, liver and thyroid function blood tests; and suicide risk assessment. Adverse drug effects were monitored. One hundred thirty-three individuals were screened online using the Schizotypal Personality Questionnaire (SPQ)^29^ to recruit those with low (<10) and high (≥40) schizotypy scores who were aged 18–45 years. Current psychiatric disorder was excluded with the Structured Clinical Interview for DSM-IV^30^. Mental state assessments included: Brief Psychiatric Rating Scale^31^, Positive and Negative Syndrome Scale (PANSS)^32^, Launay-Slade Hallucination Scale^33^ and Columbia-Suicide Severity Rating Scale (C-SSRS)^34^. Intelligence quotient was assessed with the National Reading Test^35^. One hundred five participants were randomized, 2 did not complete the study, and 96 had acceptable fMRI/cognitive task data and were included in the analysis. Participants with LS (*n* = 51) and HS (*n* = 45) received placebo (*n* = 32), ulotaront (*n* = 30; single dose, 50 mg) or amisulpride (*n* = 34; single dose, 400 mg) (dosages chosen as the highest that did not cause side effects) (Fig. 1A). Randomization was stratified by site and schizotypy according to a 1:1:1 code accessible only to pharmacy staff, producing groups with similar demographics (Fig. 1B). Participants underwent fMRI scans 2 h posttreatment. Full clinical assessments were carried out each visit and patients were asked about side effects.

### Monetary incentive delay task

Each trial consisted of a cue indicating trial type (win, loss, or neutral) followed by a target cue requiring a rapid button press, which triggered win, loss, or neutral outcomes (Fig. 1C)^36^. There were three trial types: possible reward, possible punishment, and neutral, spread across 120 trials and with a winning rate of 63%. In reward trials, fast response to the target resulted in monetary gain (win) and slow response had no consequence. In loss trials, fast response had no consequence, while slow response led to loss of money. In neutral trials, responses had no consequence. Winning and losing cues also indicated whether participants could win/lose either £1 or £5. The amount won or lost was displayed. fMRI responses were recorded in the: (1) anticipatory phase, from the initial cue to the response to the target; and (2) outcome phase, from the win/loss display.

### N-Back

Subjects viewed a series of letters and were asked to indicate if the letter presented was an “x” (0-Back) or if it matched letters shown in one (1-Back), two (2-Back) and three (3-Back) previous trials, with increasing difficulty level^17^ (see Supplementary material for full details).

### Emotional test battery

ETB tasks presented stimuli of differing emotional valence (positive/negative words or face stimuli) and tested ability to remember words and identify face emotions. Participants completed standard emotional word-based memory recall (EREC) and face-based emotional categorization (ECAT) tasks^37,38^. Instructions were presented on screen for participants to read before task completion. The psychometric properties of these tasks have been assessed previously^39^, with intraclass correlation coefficients of 0.4–0.8. Previous studies showed that antidepressant treatment led to improved identification and recall for positive relative to negative stimuli^40^.

### Functional magnetic resonance imaging (fMRI)

Scanner details and sequences, acquisition of field maps, data production and anatomical reference image acquisition, time-correction, realignment, normalization, and smoothing were carried out as described in Supplementary material. For primary region of interest (ROI) analyses, ROIs were predefined using 10-mm radius spheres centered on coordinates from a meta-analysis of MID studies^41^ (Fig. 1C) and for dorsolateral prefrontal cortex (DLPFC) in the N-Back task^16^. Anatomical masks were used to pre-define other ROIs in the N-Back task. The methodologies for RSC and ASL are described in Supplementary material.

### Statistics

A formal sample size calculation was not attempted for this 2-center fMRI MID paradigm. Based on a statistical investigation of our previous fMRI studies^42^ and the MID literature^14^ a target of 36 subjects per treatment arm was selected, requiring a sample of 108 to complete. The primary endpoints were BOLD fMRI responses in key ROIs while performing the MID, N-Back, and SD tasks after a single-dose of study medication. The ROIs are specified in the text and in Supplementary methods. The secondary endpoints were the performance measures in each task.

In the MID task, reaction time was measured in milliseconds and analyzed with repeated measures ANOVA with trial type (win, loss, neutral) as a within-subject factor, schizotypy and treatment as between-subject factors, and site and gender as covariates. After checks for equivalence of group standard deviations, analysis of variance (ANOVA) of BOLD signal task responses in ROIs (Fig. 1D) was carried out with treatment and schizotypy as fixed effects, without factors or covariates for site, task, phase or gender. Primary comparisons were placebo versus ulotaront, followed by placebo versus amisulpride and ulotaront versus amisulpride. The same design was used to analyze N-Back ROI BOLD responses. There was no correction for repeated group comparisons in several ROIs. However, in small volume family-wise error (FWE) correction, ROIs were combined into a single set of voxels to threshold statistical significance for activated voxels. Whole-brain voxels were used to threshold significant activations outside predicted ROIs.

For the N-Back task, difficulty (0, 1, 2 or 3-Back) was a within-subject factor in repeated measures ANOVAs of accuracy and reaction time. Two primary effects tested were treatment and treatment × task difficulty. Equivalent effects were tested in analyses of schizotypy and schizotypy × treatment interactions. Significant effects were followed-up by post hoc tests. BOLD signal responses in predefined ROIs contrasted all levels of task difficulty with the 0-back condition using the same ANOVA design as for the MID task.

ETB data were tested using repeated measures ANOVA (within-subject factor = positive/negative valence of stimuli and between-subject factor coded for treatment). For each task, the primary contrasts were interaction between treatment group and within-subject factors to test for emotion-specific treatment effects on performance. The primary prediction was that ulotaront would induce positive changes in ETB endpoints relative to placebo. ANOVA was also performed to determine the effects of schizotypy across treatments. Significant effects were followed-up by post hoc tests.

## Results

### MID task

ANOVA of MID task behavioral data showed participants were significantly faster on win and loss compared with neutral trials with no effect of schizotypy (Supplementary Fig. 1). Repeated measures ANCOVA of trial type × schizotypy × treatment using site and sex covariates revealed an effect of trial type [*F* (1.70,149.48) = 5.69; *p* = 0.006] with participants significantly faster on win (*p* < 0.001; CI: –16.34, –7.72) and loss (*p* < 0.001; CI: –15.51, –6.70), compared to neutral trials (Supplementary Fig. 1). There was a main effect of treatment [*F* (2,88) = 3.98, *p* = 0.02], with participants in the ulotaront group showing a significant slowing of reaction time compared to placebo (*p* = 0.049; CI: 0.9, 42.96) and amisulpride (*p* = 0.048, CI: 0.14, 42.31) groups. Nonsignificant differences were observed between placebo and amisulpride.

**Fig. 2:**
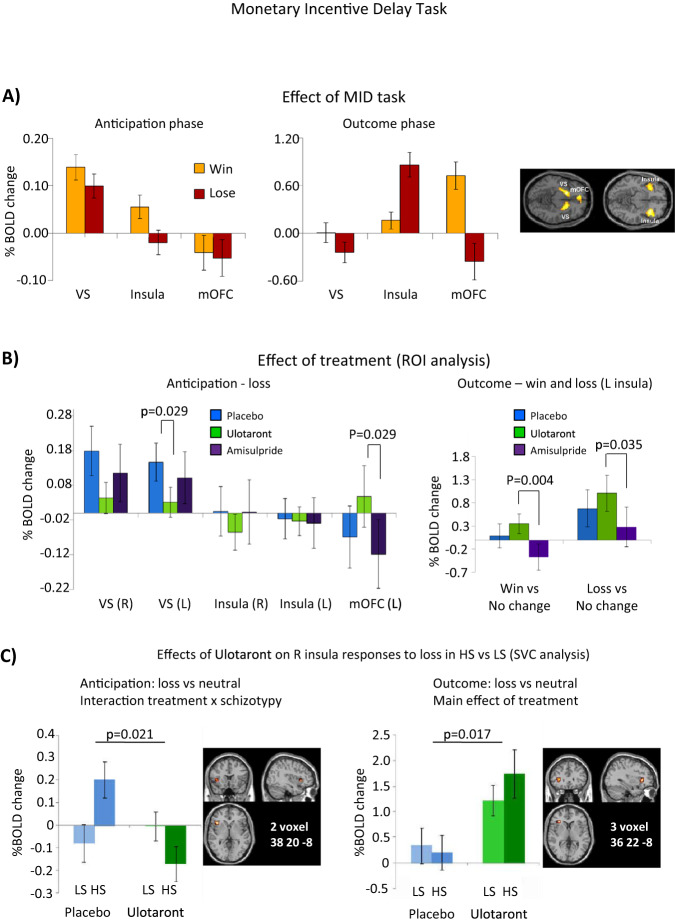
Main effects of MID task and treatment on BOLD response. **A** Main effects of MID task on ROI BOLD signals in anticipation and outcome phases in VS, insula and mOFC. **B** Effects of treatment with ulotaront, amisulpride or placebo on BOLD signals in VS, insula and mOFC ROIs during anticipation of loss, and on left insula responses in win trials in anticipation and outcome phases. **C** High schizotypes are sensitive to loss anticipation and this is reversed by ulotaront but responses to loss outcomes are similar in HS and LS and ulotaront enhanced these responses. Whole-brain SVC analysis with FWE *p* < 0.05 correction. BOLD blood oxygen level‒dependent, FWE family-wise error, HS high schizotypy, LS low schizotypy, MID monetary incentive delay, mOFC medial orbital frontal cortex, ROI region of interest, SVC small volume correction, VS ventral striatum. Error bars are standard errors of the mean.

The MID task changed BOLD signals bilaterally in prespecified ROIs (phase × valence × ROI interaction; *p* < 0.001) (Fig. 2A). During the anticipation phase, VS was activated and medial orbitofrontal cortex (mOFC) deactivated in win and loss trials (Fig. 2A). In the outcome phase, BOLD responses in insula and mOFC were substantially greater than in the anticipation phase, with mOFC responding more to win and insula to loss outcomes (Fig. 2A). There were no main effects of schizotypy in win or loss versus neutral trials.

The pattern of treatment effects on BOLD responses was similar for anticipation of wins and losses **(**see Supplementary Table 1 for all comparisons). Striatal BOLD responses were smaller in both treatment groups, and significantly so for the ulotaront group in anticipation of loss (striatum left, *p* = 0.03; right *p* = 0.06) (Fig. 2B), compared with placebo. The statistical effect of ulotaront on left or right striatal responses to loss was unaffected by including reaction time in the model as a covariate that did not approach significance. Reaction time did not correlate with left or right striatal BOLD responses (*r* < –0.11; *n* = 96). Insula responses were not significantly affected by treatment. In mOFC, win and loss anticipation evoked small negative BOLD responses that became positive in the ulotaront group in win trials compared to placebo (left and right mOFC, *p* < 0.01) (Supplementary Table 1) and in loss trials (i.e., loss-avoidance trials) compared to amisulpride (left, *p* = 0.029) (Fig. 2B); (right, *p* = 0.048) (Supplementary Table 1). In the outcome phase, there were no treatment effects in striatum or mOFC. In left insula, ulotaront increased responses to wins (*p* = 0.004) and losses (*p* = 0.035), compared to amisulpride (Fig. 2B). In right insula, HS, but not LS subjects, responded to anticipation of loss under placebo and this was prevented by ulotaront treatment**;** treatment × schizotypy was significant both in ROI (*p* = 0.021; not shown) and small volume corrected (SVC) (Fig. 2C) analyses (*p* = 0.021; FWE-corrected, *t* = 3.54). In contrast to HS responses in anticipation of potential losses, both LS and HS groups responded to loss outcomes in right insula, which was enhanced by ulotaront treatment (ROI, *p* = 0.043; SVC, *p* = 0.017) (Fig. 2C).

### N-Back

**Fig. 3:**
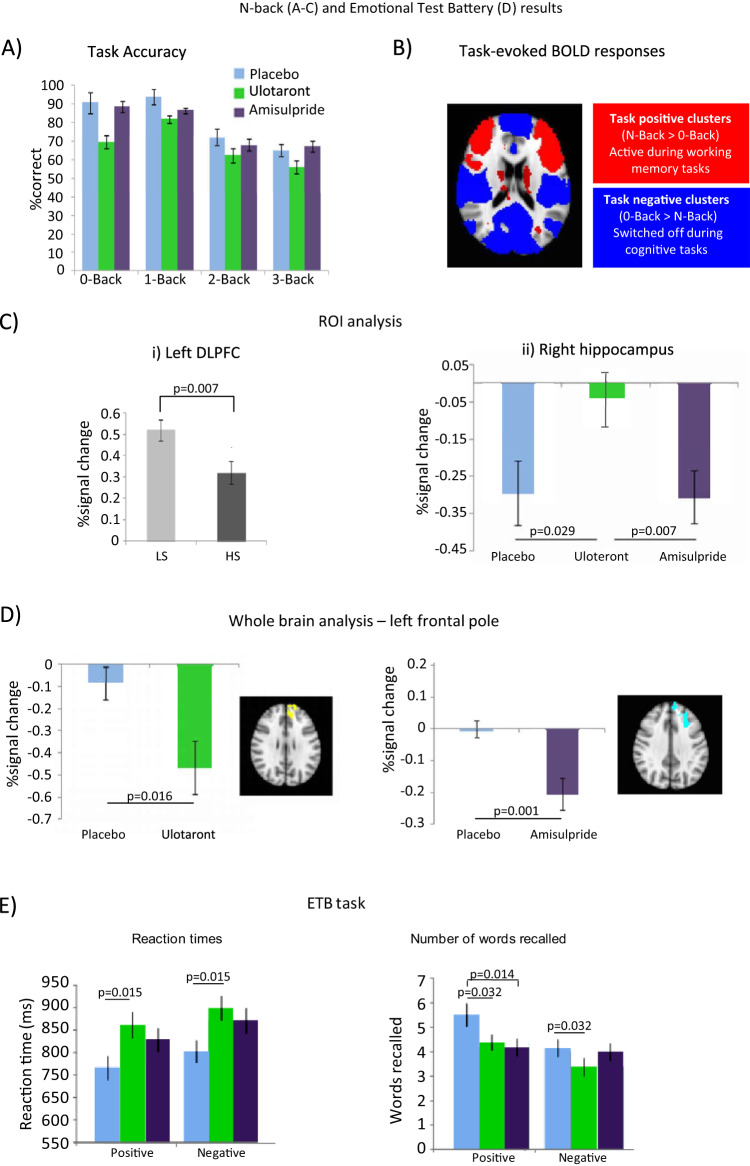
Main effects of N-Back task and treatment on BOLD response. **A** Performance at different levels of N-Back difficulty. **B** Whole-brain analysis cluster corrected *p* < 0.05 showing the main effect of task for all participants. Task-positive clusters are shown in red and task-negative clusters are in blue. **B** Main effect of treatment in hippocampus and schizotypy in DLPFC, comparing all 3 levels of N-Back with 0-Back. **C** ROI analysis showing effect of schizotypy in left DLPFC and loss of hippocampal deactivation after ulotaront treatment. **D** Whole-brain analysis showing effect of ulotaront versus placebo (–12 42 26, voxels 419) and amisulpride versus placebo (–26 38 36, voxels 729) in left frontal pole. **E** ETB analysis of emotional processing in LS and HS subjects outside the scanner and effects after treatment with placebo, ulotaront or amisulpride. The analysis assessed reaction time in the emotional categorization task and the number of both positive and negative emotional words recalled. BOLD blood oxygen level-dependent, DLPFC dorsolateral prefrontal cortex, ETB emotional test battery, HS high schizotypy, LS low schizotypy, ROI region of interest. Error bars are standard errors of the mean.

Participants receiving ulotaront were less accurate compared to placebo (*p* < 0.001) and amisulpride (*p* = 0.011) (Fig. 3A). Schizotypy did not affect accuracy. N-Back performance activated the task-positive ROIs (DLPFC, ACC and precuneus; Fig. 3B) but this was not affected by treatment. HS had smaller DLPFC BOLD responses to the task than LS participants in ROI and SVC analyses, but these were not modified by treatment (Fig. 3Ci). The expected hippocampal deactivation at all 3 levels of task difficulty was prevented on the right by ulotaront but unaffected by amisulpride (Fig. 3Cii). This was corroborated by SVC analysis (Supplementary Fig. 2). Similarly in the whole-brain analysis, de-activations in postcentral gyrus and right hippocampus were attenuated by ulotaront. Both drugs induced deactivation in left frontal pole, compared to placebo (Fig. 3D). A main effect of schizotypy showed greater activation in frontal pole in HS compared to LS groups. In 2 regions, HS versus LS differences seen under placebo were reversed in ulotaront treated participants. (Supplementary Fig. 3A–C).

### Emotional test battery

There was a main effect of treatment on reaction times in the ECAT (*p* = 0.015) (Fig. 3E). Participants receiving ulotaront (average = 882.02 ms) were slower than those receiving placebo (average = 785.21 ms). Although the effect of amisulpride versus placebo was similar, this was not significant ( > 0.10). There were no other significant treatment effects or interactions with schizotypy. In the EREC, there was an effect of treatment for total number of correct words recalled [*F* (1,55) = 4.867, *p* = 0.032]. Participants receiving ulotaront recalled fewer correct words compared to placebo (average 3.78 versus 4.83), regardless of valence (Fig. 3E). Comparing amisulpride and placebo showed an interaction between treatment and valence (*p* = 0.039; data not shown). Post hoc tests revealed participants receiving amisulpride recalled fewer correct positive words (*p* = 0.014). No differences were seen for negative words. There was an effect of schizotypy (*p* = 0.002) with HS recalling fewer words than LS participants, regardless of valence (Supplementary Tables 2 and 3).

### Resting state connectivity

**Fig. 4:**
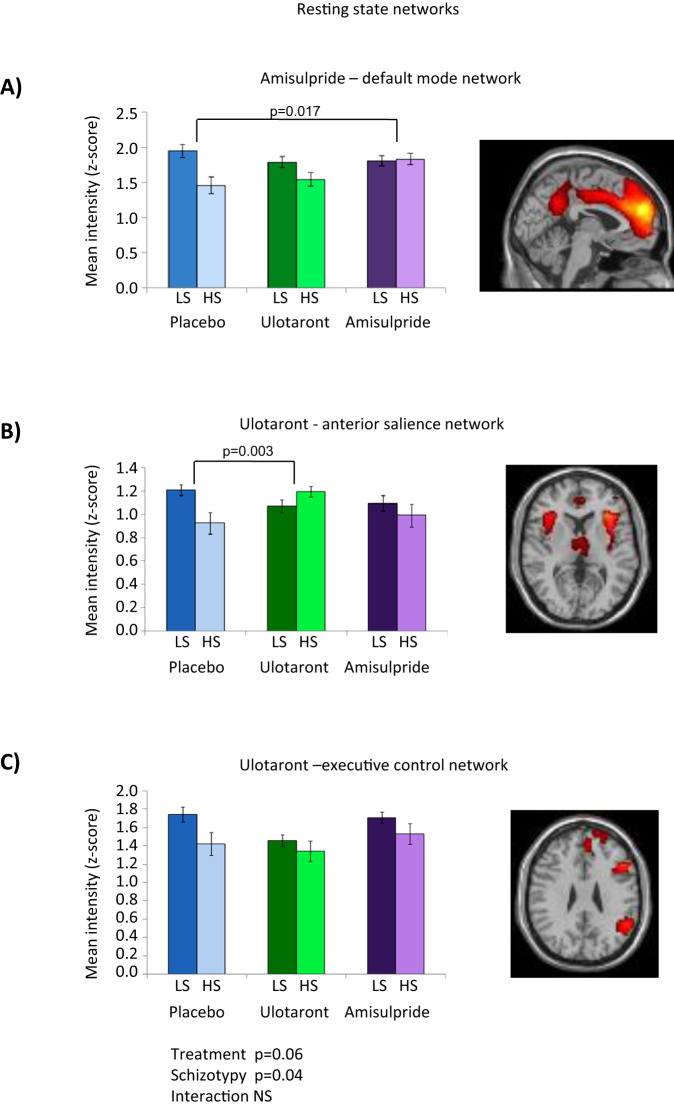
Resting state connectivity; treatment by schizotypy interactions. **A**–**C** HS group have reduced connectivity compared with LS in all networks, **A** lessened by amisulpride in DMN, **B** lessened by ulotaront in ASN, and **C** unaffected by treatment in right ECN. The histograms show group mean intensities (*z*-scores) of individual resting state networks identified from the independent component analysis. Significance values for treatment by schizotypy shown in **A** and **B** are from ANOVA with factors for treatment and schizotypy group. ASN anterior salience network, DMN default mode network, ECN executive control network. Error bars are standard errors of the mean.

Treatment × schizotypy × site ANOVA for pair-wise treatment comparisons of RSC showed a significant schizotypy effect (*p* = 0.002) with HS having significantly reduced DMN, ASN, and right ECN connectivity compared to LS participants. Both drugs tended to reduce schizotypy effects (Fig. 4). In DMN, there was a significant treatment ×schizotypy interaction, with connectivity in HS subjects under placebo increased by amisulpride to equal LS connectivity (*p* = 0.017) (Fig. 4A). In contrast, in the ASN, the effect of schizotypy was reversed by ulotaront (treatment × schizotypy, *p* = 0.003) but not amisulpride, especially in insula (Fig. 4B). A similar pattern was seen in right ECN but there were no statistically significant treatment × schizotypy interactions in whole ECN (Fig. 4C). However, the right inferior frontal cortical component of the ECN showed markedly reduced connectivity in HS participants that was abolished by ulotaront (*p* = 0.005) and by amisulpride treatment at trend level (*p* = 0.063) (Supplementary Fig. 4). In left ECN, the effect of schizotypy was not modified by ulotaront or amisulpride.

### Arterial spin labeling

**Fig. 5:**
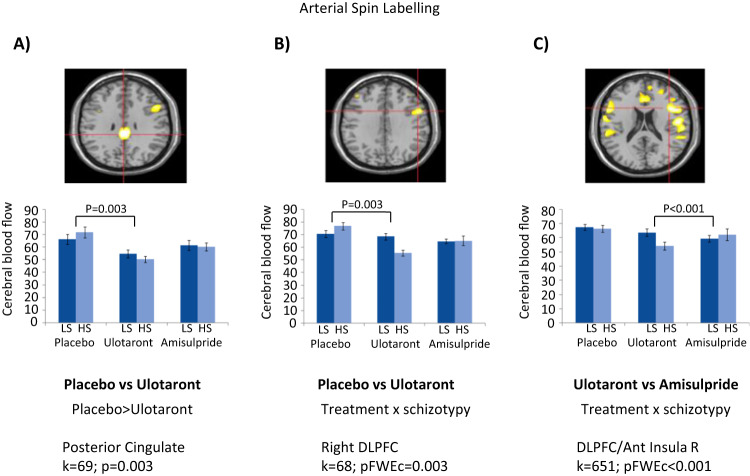
Arterial spin labeling; treatment by schizotypy interactions. Analysis of CBF for **A** placebo versus ulotaront treatment effect in the posterior cingulate, **B** Ulotaront/placebo by schizotypy interaction in right DLPFC, and **C** Ulotaront/amisulpride by schizotypy interaction in right DLPFC/insula cluster. CBF cerebral blood flow, DLPFC dorsolateral prefrontal cortex, LS low schizotypy, HS high schizotypy, pFWEc family-wise error-corrected *p*-value. Error bars are standard errors of the mean.

Ulotaront significantly reduced CBF compared to placebo in posterior cingulate (*p* = 0.003) (Fig. 5A), right thalamus (Supplementary Fig. 5A) and right DLPFC (*p* = 0.003) (Supplementary Fig. 5B). In DLPFC, greater reductions in CBF occurred in HS compared to LS participants in the ulotaront versus placebo (Fig. 5B) and ulotaront versus amisulpride (Fig. 5C) comparisons. Amisulpride-induced reductions in CBF were significant in right DLPFC and bilateral superior temporal cortex and did not differ in HS and LS participants in any region. Compared with amisulpride, ulotaront produced greater reduction in CBF in HS than the LS group in anterior cingulate (Supplementary Fig. 5C), bilateral DLPFC and insula, in right thalamus and left supramarginal gyrus. Mean CBF under placebo did not differ between HS and LS groups.

### Safety

For participants receiving ulotaront, the most commonly reported (≥10%) treatment-emergent adverse events (TEAEs) were somnolence (60.0%), nausea (34.3%), dizziness (28.6%), dry mouth (14.3%), and headache (11.4%) (Supplementary Table 4). There were no deaths or TEAEs leading to discontinuation from the study and no clinically meaningful changes in the safety parameters.

## Discussion

In summary, ulotaront partially met the primary endpoint in reducing anticipatory VS responses in the MID and reversed aberrant right insula responses to anticipated loss in HS participants. On the secondary endpoints, reductions in performance accuracy and speed were seen with ulotaront in the ETB and N-Back tasks. In HS participants, ulotaront improved reduced RSC and exerted regionally selective effects on cortical CBF.

In the MID task, the finding that participants were significantly faster on win or loss trials compared with neutral trials suggested that performance was controlled by appropriate motivational drives to win or avoid losing. The task engaged neural activity in prespecified ROIs during anticipation of monetary win or loss, consistent with previous studies^43–45^. The finding that VS responded more to anticipation of possible wins than win outcomes is consistent with the role of dopamine in prediction error learning, in which cues predicting rewards elicit anticipatory dopamine neural activity^46^. Cues allowing avoidance of aversive outcomes acquire incentive salience in the same way, albeit motivated by threat. This may account for striatal BOLD responses to cues predicting possible loss. We predicted ulotaront would attenuate striatal anticipatory BOLD responses through indirect actions in modulating dopamine neural activity. The effects of ulotaront occurred bilaterally in striatum but were significant only in anticipation of loss in left striatum. In contrast, amisulpride did not attenuate striatal responses, despite its D_2_ receptor affinity, as recently reported by Grimm et al.^47^, possibly due to competing presynaptic disinhibition of dopamine release at low doses^10^. In the MID task, ulotaront treatment achieved the primary outcome in modulating striatal responses to incentive stimuli. Psychotic symptoms in schizophrenia may be moderated by normalizing salience abnormalities in the reward system^48^. Thus, ulotaront may modulate dopaminergic incentive circuitry relevant to improvement of positive schizophrenia symptoms.

Compared with a quiescent, deactivated state under placebo, mOFC showed bilateral positive BOLD responses induced by ulotaront in win and loss anticipation. This contrasts with reduced anticipatory responses in VS following ulotaront pretreatment. These regionally contrasting effects between mOFC and VS could indicate that ulotaront lessens transfer of incentive to the cue. The action of ulotaront might normalize aberrant learning and delusion formation in hyper-dopaminergic psychotic states. No drug effects were seen in the outcome phase in mOFC, suggesting no modification of outcome value, thought to be encoded in this brain region.

We found effects of schizotypy only in insula, a key component of the salience network. Anticipation of loss activated right insula only in HS subjects and this was normalized by ulotaront. This treatment × schizotypy interaction in ROI analysis was robust to FWE correction in the SVC analysis. The differing responses may reflect aberrant salience processing in anticipation of potential loss, which was reversed by both drugs. Aberrant salience processing has been implicated in positive symptoms of schizophrenia^49^. These results in HS are consistent with evidence implicating aberrant right insula structure and function in schizophrenia pathogenesis^50–52^. In left insula, ulotaront enhanced activations to wins and losses compared with attenuations after amisulpride. In right insula, ulotaront significantly enhanced loss responses but not wins. Enhanced insula responses to outcomes could predict beneficial effects of ulotaront on affective blunting, a key component of the negative syndrome^53^.

Considering the secondary objectives, in the N-Back task, neither ulotaront nor amisulpride modulated BOLD signals in prespecified ROIs associated with cognitive function^54,55^. Ulotaront reduced hippocampal deactivation compared to placebo, and LS participants showed reduced activation in prefrontal cortex, confirming task sensitivity to schizotypy^56^. Several studies corroborate stronger negative connectivity between prefrontal cortex and right hippocampus during working memory as an endophenotype of schizophrenia, and thus reduced right hippocampal deactivation in our N-back task would be compatible with an antipsychotic action of ulotaront^57^. However, ulotaront affected task performance by reducing accuracy across several conditions, including 0-Back trials. These findings may be partially due to a high proportion of participants (60%) experiencing somnolence/sedation. The reduced hippocampal deactivation may indicate decreased ability to switch off this region of the DMN in engaging executive networks for successful task completion^58^.

In RSC analyses, HS subjects showed reduced network intensity in dorsal and ventral DMN, ASN and right ECN. Each network is defined by the functional anatomy of the voxels whose low frequency BOLD fluctuations correlate with a shared underlying time series; the correlations being taken to reflect connectivity^59,60^. Recent studies suggested that schizophrenia is characterized by reduced connectivity within these networks^52,61–63^. HS participants generally showed reduced connectivity compared to LS under placebo, and both ulotaront and amisulpride reduced or abolished LS-HS differences with some selectivity for different networks. Ulotaront was effective in reversing decreased connectivity in HS participants in ASN (principally bilateral insula) but not significantly in DMN or ECN, whereas amisulpride was effective selectively in DMN. The selective effect of ulotaront in HS subjects in insula was remarkably convergent with the MID task finding that ulotaront reduced aberrant responses to losses in HS participants in this ROI and in keeping with other studies indicating a key role of insula in schizophrenia dysconnectivity^52,62,64^. Notwithstanding the interest of these results, it should be noted that there is considerable uncertainty about the unitary nature of schizotypy in the general population, how it is best measured, and its relationship to psychosis risk^9^. We suggest that the utility of cognition in schizotypy as a surrogate for psychosis in drug-development remains experimental.

There was little evidence of a schizotypy effect on regional CBF. Both ulotaront and amisulpride tended to reduce CBF in similar ROIs in LS and HS subjects. However, ulotaront treatment reduced CBF in DLPFC, posterior cingulate, insula and other regions to a greater extent in HS than LS subjects, producing significant interactions with schizotypy compared to placebo or amisulpride. This suggested ulotaront differentially affects CBF in HS subjects. Combined with RSC data, it appears that ulotaront and amisulpride affect CBF in structures prominent in overlapping networks, such as anterior cingulate (DMN), insula (ASN) and DLPFC (ECN). In each region, ulotaront reduced connectivity and CBF more in HS than LS subjects, suggesting it may engage processes underlying HS, and potentially those in schizophrenia. In contrast, neither ulotaront nor amisulpride showed an antidepressant-like profile on the ETB.

There are limitations to the current study. The investigation was designed to profile effects of ulotaront across a range of cognitive and connectivity biomarkers relevant to schizophrenia and to the antipsychotic and antidepressant behavioral profiles detected in preclinical development^1^. There is a problem of multiple comparisons inherent in profiling and maximizing the information extracted. This is exacerbated by analyzing factors other than treatment that could influence performance, including site, sex, schizotypy, task, behavioral measures, and ROIs. We attempted to mitigate this by selecting a primary outcome measure (decreased VS response in reward/loss anticipation) and powering sample size to 36/treatment arm. The study was not designed or powered to detect efficacy on a limited number of exploratory endpoints but rather to profile the effects of the new drug, ulotaront, on a range of measures of potential relevance to clinical development for schizophrenia.

The interpretation of some results is complicated by drowsiness reported by 60% of those taking ulotaront and their reduced response speed and accuracy in some of the tasks. In the MID task, minimally slower responses of the ulotaront group did not prevent the speeding induced by the prospect of winning and not losing and this suggests a full engagement in the task. Furthermore, the reduced activation in striatum after ulotaront was not due to the 8% increase in reaction time since it was unaffected by covarying reaction time in the ANOVA. In the N-Back task, covarying for poorer accuracy did not abolish the drug effects on fMRI responses in a post hoc analysis (data not shown). These mitigations notwithstanding, drug-induced sedation remains a potential confound in the task-evoked responses. However, ulotaront reversed the effect of HS versus LS in the insula both on BOLD responses to loss anticipation in the MID task, and in insula connectivity within the salience network. Differential HS versus LS effects of ulotaront were also seen in non-ROI regions in the N-Back task and in RSC. These HS selective effects would seem relatively immune from the confound of drowsiness.

In conclusion, the results indicated that ulotaront’s ability to reduce presynaptic dopamine function in preclinical development translated to phase 1 effects in modifying dopamine-related fMRI responses in the MID task. Furthermore, ulotaront reduced the effects of HS in insula in the MID and RSC and in other regions in the N-Back task. This translational evidence of functional target engagement by ulotaront together with the absence of effects in the ETB, led to phase 2 studies prioritizing efficacy trials in schizophrenia. Antipsychotic efficacy was subsequently reported in a phase 2 clinical trial (4) and ulotaront is currently undergoing evaluation for treatment of schizophrenia in randomized controlled phase 3 clinical trials.

### Supplementary information

2022TP001586R Supplementary material

## Data Availability

Access to de-identified participant data will be provided after a research proposal is submitted online (https://vivli.org) and receives approval from the Independent Review Panel and after a data sharing agreement is in place. Access will be provided for an initial period of 12 months after approval of the data sharing request, but an extension can be granted, when justified, for up to an additional 12 months.

## References

[1] Dedic N (2019). SEP-363856, a novel psychotropic agent with a unique, non-D(2) receptor mechanism of action. J. Pharmacol. Exp. Ther..

[2] Dedic N, Dworak H, Zeni C, Rutigliano G, Howes OD (2021). Therapeutic potential of TAAR1 agonists in schizophrenia: evidence from preclinical models and clinical studies. Int. J. Mol. Sci..

[3] Kokkinou M (2021). Reproducing the dopamine pathophysiology of schizophrenia and approaches to ameliorate it: a translational imaging study with ketamine. Mol. Psychiatry.

[4] Hopkins SC, Dedic N, Koblan KS (2021). Effect of TAAR1/5-HT(1A) agonist SEP-363856 on REM sleep in humans. Transl. Psychiatry.

[5] Koblan KS (2020). A non-D2-receptor-binding drug for the treatment of schizophrenia. N. Engl. J. Med..

[6] Correll CU (2021). Safety and effectiveness of ulotaront (SEP-363856) in schizophrenia: results of a 6-month, open-label extension study. NPJ Schizophr..

[7] Vu MA (2013). Working memory in schizotypal personality disorder: fMRI activation and deactivation differences. Schizophr. Res..

[8] Ettinger U, Meyhöfer I, Steffens M, Wagner M, Koutsouleris N (2014). Genetics, cognition, and neurobiology of schizotypal personality: a review of the overlap with schizophrenia. Front. Psychiatry.

[9] Lenzenweger MF (2015). Thinking clearly about schizotypy: hewing to the schizophrenia liability core, considering interesting tangents, and avoiding conceptual quicksand. Schizophr. Bull..

[10] McKeage K, Plosker GL (2004). Amisulpride: a review of its use in the management of schizophrenia. CNS Drugs.

[11] Juckel G (2006). Dysfunction of ventral striatal reward prediction in schizophrenia. Neuroimage.

[12] Juckel G (2006). Dysfunction of ventral striatal reward prediction in schizophrenic patients treated with typical, not atypical, neuroleptics. Psychopharmacology (Berl).

[13] Papalini S (2019). The predictive value of neural reward processing on exposure therapy outcome: Results from a randomized controlled trial. Prog. Neuropsychopharmacol. Biol. Psychiatry.

[14] Heinz A, Schlagenhauf F (2010). Dopaminergic dysfunction in schizophrenia: salience attribution revisited. Schizophr. Bull..

[15] Kirschner M (2018). Ventral striatal dysfunction and symptom expression in individuals with schizotypal personality traits and early psychosis. Schizophr. Bull..

[16] Owen AM, McMillan KM, Laird AR, Bullmore E (2005). N-back working memory paradigm: a meta-analysis of normative functional neuroimaging studies. Hum. Brain Mapp..

[17] Koychev I (2012). A validation of cognitive biomarkers for the early identification of cognitive enhancing agents in schizotypy: a three-center double-blind placebo-controlled study. Eur. Neuropsychopharmacol.

[18] Harmer CJ (2003). Acute SSRI administration affects the processing of social cues in healthy volunteers. Neuropsychopharmacology.

[19] Harmer CJ, Hill SA, Taylor MJ, Cowen PJ, Goodwin GM (2003). Toward a neuropsychological theory of antidepressant drug action: increase in positive emotional bias after potentiation of norepinephrine activity. Am. J. Psychiatry.

[20] Harmer CJ, Shelley NC, Cowen PJ, Goodwin GM (2004). Increased positive versus negative affective perception and memory in healthy volunteers following selective serotonin and norepinephrine reuptake inhibition. Am. J. Psychiatry.

[21] Harmer CJ (2009). Effect of acute antidepressant administration on negative affective bias in depressed patients. Am. J. Psychiatry.

[22] Hadley JA (2016). Change in brain network topology as a function of treatment response in schizophrenia: a longitudinal resting-state fMRI study using graph theory. NPJ Schizophr..

[23] MacKay MB (2018). Multidimensional connectomics and treatment-resistant schizophrenia: linking phenotypic circuits to targeted therapeutics. Front. Psychiatry.

[24] Menon V (2011). Large-scale brain networks and psychopathology: a unifying triple network model. Trends Cogn. Sci..

[25] Mikolas P (2016). Connectivity of the anterior insula differentiates participants with first-episode schizophrenia spectrum disorders from controls: a machine-learning study. Psychol. Med..

[26] Rodriguez M (2019). Cognitive profiles and functional connectivity in first-episode schizophrenia spectrum disorders - linking behavioral and neuronal data. Front. Psychol..

[27] Schneider K (2019). Cerebral blood flow in striatal regions is associated with apathy in patients with schizophrenia. J. Psychiatry Neurosci..

[28] Kindler J (2018). Increased striatal and reduced prefrontal cerebral blood flow in clinical high risk for psychosis. Schizophr. Bull..

[29] Raine A (1991). The SPQ: a scale for the assessment of schizotypal personality based on DSM-III-R criteria. Schizophr. Bull..

[30] First, M. B., Williams, J. B. W., Spitzer, R. L. & Gibbon, M. *Structured Clinical Interview for DSM-IV-TR Axis I Disorders, Clinical Trials Version (SCID-CT)* (American Psychiatric Publishing, Inc.; Washington, DC, 2007).

[31] Overall JE, Gorham DR (1962). The Brief Psychiatric Rating-scale. Psychol. Rep..

[32] Kay SR, Fiszbein A, Opler LA (1987). The positive and negative syndrome scale (PANSS) for schizophrenia. Schizophr. Bull..

[33] Launay G, Slade P (1981). The measurement of hallucinatory predisposition in male and female prisoners. Pers. Individ. Dif..

[34] Posner K (2011). The Columbia-Suicide Severity Rating Scale: initial validity and internal consistency findings from three multisite studies with adolescents and adults. Am. J. Psychiatry.

[35] Conoley J. C. & Impara J. C. *The Twelfth Mental Measurements Year Book*, 12th edn. Buros (Institute of Mental Measurements, 1995).

[36] Knutson B, Fong GW, Adams CM, Varner JL, Hommer D (2001). Dissociation of reward anticipation and outcome with event-related fMRI. Neuroreport.

[37] Browning M, Reid C, Cowen PJ, Goodwin GM, Harmer CJ (2007). A single dose of citalopram increases fear recognition in healthy subjects. J. Psychopharmacol.

[38] Browning M (2019). Predicting treatment response to antidepressant medication using early changes in emotional processing. Eur. Neuropsychopharmacol.

[39] Thomas JM, Higgs S, Dourish CT (2016). Test-retest reliability and effects of repeated testing and satiety on performance of an Emotional Test Battery. J. Clin. Exp. Neuropsychol..

[40] Arnone D, Horder J, Cowen PJ, Harmer CJ (2009). Early effects of mirtazapine on emotional processing. Psychopharmacology (Berl).

[41] Liu X, Hairston J, Schrier M, Fan J (2011). Common and distinct networks underlying reward valence and processing stages: a meta-analysis of functional neuroimaging studies. Neurosci. Biobehav. Rev.

[42] Goodwin, G. M. et al. *Effects of Cognitive and Neural Negative Biases Present in Dysphoric Volunteers on an Emotional Test Battery and the Associated Modulation of fMRI BOLD Signals [poster]*. Presented at American College of Neuropsychopharmacology, December 4–9, 2010, Miami Beach, FL (2010).

[43] Knutson B, Westdorp A, Kaiser E, Hommer D (2000). fMRI visualization of brain activity during a monetary incentive delay task. Neuroimage.

[44] Knutson B, Bhanji JP, Cooney RE, Atlas LY, Gotlib IH (2008). Neural responses to monetary incentives in major depression. Biol. Psychiatry.

[45] Oldham S (2018). The anticipation and outcome phases of reward and loss processing: a neuroimaging meta-analysis of the monetary incentive delay task. Hum. Brain. Mapp..

[46] Schultz W (1997). Dopamine neurons and their role in reward mechanisms. Curr. Opin. Neurobiol..

[47] Grimm O (2021). No effect of a dopaminergic modulation fMRI task by amisulpride and L-DOPA on reward anticipation in healthy volunteers. Psychopharmacology (Berl).

[48] Wulff S (2020). The relation between dopamine D(2) receptor blockade and the brain reward system: a longitudinal study of first-episode schizophrenia patients. Psychol. Med..

[49] Kapur S (2003). Psychosis as a state of aberrant salience: a framework linking biology, phenomenology, and pharmacology in schizophrenia. Am. J. Psychiatry.

[50] Modinos G (2013). Neuroanatomy of auditory verbal hallucinations in schizophrenia: a quantitative meta-analysis of voxel-based morphometry studies. Cortex.

[51] Peters SK, Dunlop K, Downar J (2016). Cortico-striatal-thalamic loop circuits of the salience network: a central pathway in psychiatric disease and treatment. Front. Syst. Neurosci..

[52] Luo Q (2020). Effective connectivity of the right anterior insula in schizophrenia: the salience network and task-negative to task-positive transition. Neuroimage Clin..

[53] Hornix BE, Havekes R, Kas MJH (2019). Multisensory cortical processing and dysfunction across the neuropsychiatric spectrum. Neurosci. Biobehav. Rev..

[54] Feifel D, Shilling PD, MacDonald K (2016). A review of oxytocin’s effects on the positive, negative, and cognitive domains of schizophrenia. Biol. Psychiatry.

[55] Mars RB (2012). On the relationship between the "default mode network" and the "social brain". Front. Hum. Neurosci..

[56] Vatansever D, Menon DK, Manktelow AE, Sahakian BJ, Stamatakis EA (2015). Default mode dynamics for global functional integration. J. Neurosci..

[57] Schneider M (2017). Altered DLPFC-hippocampus connectivity during working memory: independent replication and disorder specificity of a putative genetic risk phenotype for schizophrenia. Schizophr. Bull..

[58] Carrigan N, Barkus E, Ong A, Wei M (2017). Do complaints of everyday cognitive failures in high schizotypy relate to emotional working memory deficits in the lab?. Compr. Psychiatry.

[59] Li X (2019). Clinical utility of the dual n-back task in schizophrenia: a functional imaging approach. Psychiatry Res. Neuroimaging.

[60] Cordes D (2001). Frequencies contributing to functional connectivity in the cerebral cortex in "resting-state" data. AJNR Am. J. Neuroradiol..

[61] Zhu J, Zhu DM, Qian Y, Li X, Yu Y (2018). Altered spatial and temporal concordance among intrinsic brain activity measures in schizophrenia. J. Psychiatr. Res..

[62] Hilland E (2022). Aberrant default mode connectivity in adolescents with early-onset psychosis: a resting state fMRI study. Neuroimage Clin..

[63] Gradin VB (2013). Salience network-midbrain dysconnectivity and blunted reward signals in schizophrenia. Psychiatry Res..

[64] Manoliu A (2014). Aberrant dependence of default mode/central executive network interactions on anterior insular salience network activity in schizophrenia. Schizophr. Bull..

