# Supplementary material for: Effects of ulotaront on brain circuits of reward, working memory, and emotion processing in healthy volunteers with high or low schizotypy
Source: Schizophrenia (Heidelb). 2023 Aug 7;9(1):49. doi: 10.1038/s41537-023-00385-6 (PMC10406926; doi:10.1038/s41537-023-00385-6)

Supplementary material

Method of Assigning Subjects to Treatment Groups (From the statistical analysis plan)
Subjects will be randomised to the 3 study drug treatment arms, using a pre-determined randomisation schedule, in the ratio of 1:1:1. Subjects will be stratified by site and schizotypy (based on screening SPQ score). Each treatment arm will consist of 2 groups with either low or high schizotypy (based on Visit 1 SPQ scores) for a total of 6 groups.

- Group 1: High schizotypy - SEP-363856 (single-dose 50 mg)
- Group 2: Low schizotypy - SEP-363856(single-dose 50 mg)
- Group 3: High schizotypy - Amisulpride (single-dose 400 mg)
- Group 4: Low schizotypy - Amisulpride (single-dose 400 mg)
- Group 5: High schizotypy - Placebo.
- Group 6: Low schizotypy - Placebo

Blinding (From the statistical analysis plan)
This is a double-blind study with an open-label pharmacy. Subjects, Investigator staff (except pharmacy), persons performing the assessments, clinical operations personnel (except unblinded monitors), data analysts, and the personnel at central laboratories (including imaging) remained blinded to the identity of the treatment from the time of randomization until database lock and unblinding. Randomization data were kept confidential and were not accessible to anyone involved in the study except the following: pharmacy staff, interim analysis team members and safety data review team members or Data Monitoring Committee (DMC) members involved in the interim data review.

Criteria for Evaluation (Protocol version 2, EudraCT No. 2013-003801-24)**:**
Primary Endpoint: BOLD fMRI activity in key ROIs while performing the MID, N-back, and SD tasks after a single dose of study medication.

- BOLD fMRI activity within the ventral striatum, including the nucleus accumbens while performing win, neutral and lose trials in the MID task.
- BOLD fMRI activity within the prefrontal cortex, precuneus, hippocampus and anterior cingulate during the 0 (control), 1, 2 and 3 back conditions of the N-back task.
- BOLD fMRI activity within the auditory cortex and prefrontal regions while performing the SD task.

Secondary Endpoints:

- Behavioural performance in the MID task, includes measurements of trial accuracy, trial duration, trial reaction time, and amount of money won.
- Behavioural performance in the N-back task, includes measurements of accuracy (% correct for each trial type), reaction time and target sensitivity (d').
- Behavioural performance in the SD task, includes measurements of correct and incorrect responses.

Other Endpoints:

- BOLD fMRI signals in other brain regions while performing the MID, N-back, and SD tasks.
- Resting state BOLD fMRI signals in brain areas associated with positive, negative and cognitive symptoms.
- ASL measurements of perfusion level (absolute inflow of arterial blood).

- Behavioural performance in the ETB
    - FERT: % accuracy for each emotion, % misclassifications for each emotion, reaction time for correct answers in each emotion, reaction time for all emotions, target sensitivity for each emotion, response bias for each emotion.
    - ECAT: % accuracy for each condition, reaction time for each condition.
    - EREC: number of words recalled for each characteristic, number of commission errors.
    - FDOT: % accuracy, average reaction time and average vigilance scores for correct answers for each emotion and condition.
    - EMEM: % accuracy, % false alarms, average reaction time, target sensitivity and response bias for each characteristic.
- Change from baseline in BPRS at Visit 2.

Interim analysis

The protocol included an independent interim analysis after approximately 50% recruitment to guide planning for other studies of ulotaront with no bearing on the continuation of the present study.

<u>Task descriptions and analysis</u>

N-Back task

Subjects were shown a series of letters and asked to indicate if the letter presented was an “x” (0-Back) or if it matched letters shown in one (1-Back), two (2-Back) and three (3-Back) previous trials with increasing difficulty level. A series of alphabet letters were presented one at a time on a color monitor. Participants were instructed not to respond until they saw the same letter twice following one another. The task had three levels of difficulty according to the number of letters in between the two matching letters. In the 1-back test the two letters followed each other immediately. In the 2-back test the target letters were separated by one letter and in the three back two letters separated the target letters. Thus, participants had to hold in mind one, two, or three letters. Consequently, the 1-back test exerted the lowest load on working memory and the 3-back task the highest. 0-back blocks controlled for attending to the task where participants simply needed to respond when they saw the letter “x.” There were 4 blocks of 10 trials for each condition (0, 1, 2, or 3-back) presented in a fixed pseudorandom order.

fMRI analysis

Echo-planar T2*-weighted images were acquired with a Siemens 3T-TIM Trio scanner with body transmit and 12-channel head receive coils (Oxford) ora Philips 3-Tesla Achieva MRI scanner and 8 channel coil (Manchester). Data were generated using a 3 x 3 x 3.5mm voxel resolution; 2000ms repetition time (TR); 28ms inversion/echo time; and 87° flip angle. Thirty-seven 3mm slices were acquired, descending sequentially parallel to the anterior-posterior commissural line. Data were collected in 315 volumes/2 runs with two 636s dummy scans/run. Field maps were acquired using a dual 2D gradient-echo with echoes at 5.19 and 7.65ms (444ms repetition time). Data were produced using a 64 x 64 x 40 grid with 3mm isotropic voxel resolution. Anatomical reference images were acquired in ascending slice order within 356s using a magnetisation-prepared rapid gradient-echo sequence with 0.78 x 0.8 x 0.78mm voxel resolution on a 208 x 256 x 200 grid, and TE/TI/TR=4.8/1100/2040ms.

fMRI data processing

Statistical Parametric Mapping (SPM8) was used to analyse fMRI data. Images were corrected for time differences, realigned, normalized and smoothed with an 8mm Gaussain kernel. Artifact Detection Tools (ART) from the NeuroImaging Tools and Resources Collaboratory was used to detect artifacts in time-series data. Motion regressors were used in first level analysis. Participants with more than 15% outlying volumes were excluded. Image data were converted to Neuroimaging Informatics Technology Initiative format and preprocessed using SPM or FMRIB Software Library (FSL) analysis packages. Images were realigned using a least squares approach and 6-parameter rigid body spatial translation. A representative realigned image was used to derive parameters for spatial normalisation to the Montreal Neurological Institute standard template. To discriminate between task/intervention-related effect and noise, signals were fitted to a general linear model and β-values used to construct probability maps to give a parameter-estimate image for each subject and task condition. The outcome was an estimated contrast coefficient β and standard error (% BOLD signal change) from time-series analyses for each voxel. Inputs for primary second level ROI analyses were the weighted mean β per subject, task and ROI. In addition, whole brain inspection with a small-volume correction (SVC) family-wise error threshold of $p<0.05$ and correction for 6 ROI was carried out. For the MID task, event-related design was carried out and events of interest were divided into anticipatory and outcome phases/trial. The contrasts win/neutral and loss/neutral were modelled for the anticipatory and outcome phases. The contrast for the N-back was 1,2 and 3 back versus 0-back.

Resting state (RS) fMRI analysis

20 spatially independent components were extracted using independent component analysis (ICA) using the Group ICA of fMRI toolbox (GIFT). Components that spatially correlated with highest r for dorsal (d) and ventral (v) default mode network (DMN) masks from Stanford University were extracted and analyzed further. Component 18 had the highest correlation to the dDMN mask (r=0.56) and component 6 had the highest correlation tovDMN mask (r=0.26; Figure 9.7.6.1). Intensity maps for each component were reconstructed for each individual and the resulting maps entered into a three 3-way (treatment x schizotypy x site) ANOVA for each pair-wise treatment comparison (ulotaront vs. placebo, ulotaront vs. amisulpride and amisulpride vs. placebo).A 3-level analysis was carried out: 1) ROI analysis, in which the mean intensity value per individual was extracted for the entire component/network; 2) small volume correction (SVC) using a mask of the entire network: and 3) whole brain voxel-wise analysis where cluster-wise inference was applied. The same ROI approach was used for the salience and executive networks.

Supplementary Table 1. Monetary Incentive Delay task. Planned pairwise ulotaront vs placebo and amisulpride vs placebo contrast estimates, standard errors (se) and significance levels (p) in ROI analysis. ***Bold italic*** **p<0.05**; *Italic p≤0.10*

ANTICIPATION

**win-neutral**	ULO - Pbo	se	AMI - Pbo	se	pULO - Pbo	pAMI - Pbo	pULO - AMI
L striatum	-0.07	0.07	-0.04	0.08	0.30	0.68	0.65
R striatum	-0.07	0.07	-0.05	0.09	0.27	0.61	0.74
L insula	0.08	0.06	0.06	0.07	0.18	0.36	0.84
R insula	-0.01	0.07	-0.04	0.09	0.90	0.65	0.72
L mOFC	0.25	0.10	0.15	0.10	***0.01***	0.12	0.30
R mOFC	0.24	0.09	0.13	0.09	***0.01***	0.18	0.19

**loss -neutral**	ULO - Pbo	se	AMI - Pbo	se	pULO - Pbo	pAMI - Pbo	pULO - AMI
L striatum	-0.13	0.06	-0.06	0.07	***0.03***	0.39	0.38
R striatum	-0.12	0.07	-0.09	0.08	*0.06*	0.29	0.66
L insula	0.00	0.06	-0.01	0.08	0.99	0.94	0.94
R insula	-0.10	0.07	-0.02	0.09	0.16	0.81	0.42
L mOFC	0.19	0.10	-0.06	0.11	*0.07*	0.61	***0.03***
R mOFC	0.17	0.10	-0.05	0.10	*0.10*	0.64	***0.05***

OUTCOME

**gain-neutral**	ULO - Pbo	se	AMI - Pbo	se	pULO - Pbo	pAMI - Pbo	pULO - AMI
L striatum	0.56	0.32	-0.05	0.40	*0.09*	0.90	0.11
R striatum	0.39	0.31	-0.04	0.39	0.22	0.92	0.23
L insula	0.44	0.27	-0.45	0.28	0.12	0.13	***0.01***
R insula	0.60	0.31	-0.09	0.39	*0.06*	0.82	*0.09*
L mOFC	-0.50	0.45	-0.60	0.44	0.27	0.20	0.89
R mOFC	-0.33	0.43	-0.56	0.42	0.45	0.18	0.62

**loss -neutral**	ULO - Pbo	se	AMI - Pbo	se	pULO - Pbo	pAMI - Pbo	pULO - AMI
L striatum	0.50	0.38	0.20	0.35	0.16	0.60	0.44
R striatum	0.47	0.37	0.27	0.36	0.20	0.48	0.57
L insula	0.62	0.43	-0.35	0.41	0.14	0.42	***0.04***
R insula	0.90	0.54	0.06	0.43	***0.04***	0.91	0.13
L mOFC	-0.57	0.50	-0.08	0.66	0.39	0.87	0.40
R mOFC	-0.39	0.50	-0.28	0.64	0.54	0.58	0.82

Supplementary Table 2. Emotional Test Battery, Emotional Categorization Task. Planned pairwise drug treatment comparisons of reaction times showing significant increase in reaction times for ulotaront vs placebo and trend significant effect of Amisulpride independent of valence.

**Task and contrast**	**Post hoc test**	**Ulotaront vs. placebo**			**Ulotaront vs. amisulpride**			**Amisulpride vs. placebo**		
**EREC Task**		Estimate (95% CI)	SE	p-value	Estimate (95% CI)	SE	p-value	Estimate (95% CI)	SE	p-value
**Words recalled, Main effect of treatment**		-1.04, (-1.99 , -0.10)	0.47	**0.03**	-0.19, (-1.00 , 0.62)	0.41	0.64	-0.85, (-1.73 , 0.04)	0.44	.06
**Words recalled, Treatment x word valence interaction**				0.44			0.15			.04
	**Word recalled positive**	-1.25, (-2.39 , -0.11)	0.57	**0.03**	0.23, (-0.75 , 1.20)	0.49	0.64	-1.43, (-2.57 , -0.30)	0.57	.01
	**Words recalled Negative**	-0.84, (-1.87 , 0.20)	0.51	0.11	-0.61, (-1.62 , 0.40)	0.50	0.23	-0.26, (-1.20 , 0.68)	0.47	.58
**Commission errors, Main effect of treatment**		-0.54, (-1.51 , 0.43)	0.48	.27	-0.45, (-1.33 , 0.43)	0.44	0.31	-0.15, (-1.08 , 0.78)	0.47	.75
**Commission errors, Treatment x word valence interaction**				0.09			0.13			.55
	**Commission errors positive**	-1.03, (-2.37 , 0.30)	0.67	0.13	-0.81, (-1.97 , 0.35)	0.58	0.17	-0.32, (-1.63 , 1.00)	0.66	.63
	**Commission errors negative**	-0.04, (-0.90 , 0.82)	0.43	0.92	-0.09, (-0.89 , 0.71)	0.40	0.82	0.01, (-0.76 , 0.79)	0.39	.97

CI = confidence interval, EREC = emotional recall, SE = standard error. Bold values are significant for p<.05 (corrected).

Supplementary Table 3. Emotional Test Battery. Effect of treatment on ETB words recalled showing significant effects for ulotaront vs placebo and amisulpride vs placebo.

**Task and contrast**	**Post hoc test**	**Ulotaront vs. placebo**			**Ulotaront vs. amisulpride**			**Amisulpride vs. placebo**		
**EREC Task**		Estimate (95% CI)	SE	p-value	Estimate (95% CI)	SE	p-value	Estimate (95% CI)	SE	p-value
**Words recalled, Main effect of treatment**		-1.04, (-1.99 , -0.10)	0.47	**0.03**	-0.19, (-1.00 , 0.62)	0.41	0.64	-0.85, (-1.73 , 0.04)	0.44	.06
**Words recalled, Treatment x word valence interaction**				0.44			0.15			.04
	**Word recalled positive**	-1.25, (-2.39 , -0.11)	0.57	**0.03**	0.23, (-0.75 , 1.20)	0.49	0.64	-1.43, (-2.57 , -0.30)	0.57	.01
	**Words recalled Negative**	-0.84, (-1.87 , 0.20)	0.51	0.11	-0.61, (-1.62 , 0.40)	0.50	0.23	-0.26, (-1.20 , 0.68)	0.47	.58
**Commission errors, Main effect of treatment**		-0.54, (-1.51 , 0.43)	0.48	.27	-0.45, (-1.33 , 0.43)	0.44	0.31	-0.15, (-1.08 , 0.78)	0.47	.75
**Commission errors, Treatment x word valence interaction**				0.09			0.13			.55
	**Commission errors positive**	-1.03, (-2.37 , 0.30)	0.67	0.13	-0.81, (-1.97 , 0.35)	0.58	0.17	-0.32, (-1.63 , 1.00)	0.66	.63
	**Commission errors negative**	-0.04, (-0.90 , 0.82)	0.43	0.92	-0.09, (-0.89 , 0.71)	0.40	0.82	0.01, (-0.76 , 0.79)	0.39	.97

CI = confidence interval, EREC = emotional recall, SE = standard error. Bold values are significant for p<.05 (corrected).

Supplementary Table 4. Treatment-emergent adverse events in the safety population[a].

Characteristic, n (%)	Placebo (n=34)	Ulotaront (n=35)	Amisulpride (n=36)
At least 1 TEAE	16 (47.1)	34 (97.1)	15 (41.7)
Treatment-related TEAE[b]	13 (38.2)	33 (94.3)	12 (33.3)
Serious TEAE	0	1 (2.9)	0
TEAEs occurring in ≥5% of patients in any treatment group			
Anxiety	0	0	3 (8.3)
Disturbance in attention	1 (2.9)	0	2 (5.6)
Dizziness	2 (5.9)	10 (28.6)	1 (2.8)
Dry mouth	0	5 (14.3)	0
Fatigue	4 (11.8)	1 (2.9)	0
Headache	7 (20.6)	4 (11.4)	1 (2.8)
Hot flush	0	2 (5.7)	0
Lethargy	0	3 (8.6)	2 (5.6)
Nasopharyngitis	2 (5.9)	0	1 (2.8)
Nausea	0	12 (34.3)	0
Oropharyngeal pain	2 (5.9)	0	0
Presyncope	0	2 (5.7)	0
Sedation	0	3 (8.6)	0
Somnolence	5 (14.7)	21 (60.0)	5 (13.9)
Urinary tract infection	1 (2.9)	2 (5.7)	1 (2.8)
Vomiting	0	3 (8.6)	0

[a]All subjects who were randomized and received study drug; [b]Includes TEAEs that were considered by the investigator to have a definite, probable, possible, or missing relationship to study drug.
TEAE, treatment-emergent adverse event.

Supplementary Fig. 1. Monetary Incentive Delay task. Reaction times by high and low schizotypy (HS, LS), drug treatment and trial type: win (left block) neutral (middle block) and loss anticipation (right block). Dotted line shows overall mean reaction time for win neutral and loss trials. Signalled wins and losses evoke significantly faster responses than neutral cues. Error bars are standard errors of the mean.

Trial type p=0.006. Treatment p=0.02 (ulotaront > placebo = amisulpride).

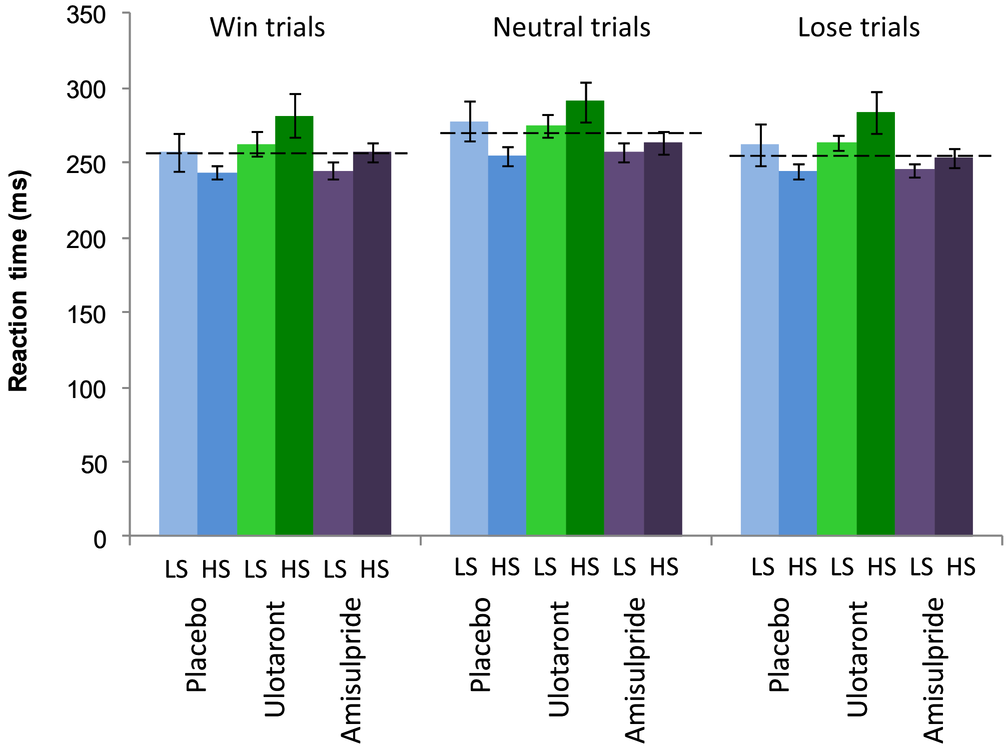

Supplementary Fig. 2. N-Back task. Small volume correction analysis confirming main effect of treatment in the ROI analysis with right hippocampal (26 -20 -18, voxels 197) deactivation prevented more in the ulotaront group than in the amisulpride. The effect of schizotypy was also confirmed in the DLPFC (-42 10 22, voxels 90). AMI = amisulpride; DLPFC = dorsolateral prefrontal cortex; HS = high schizotypy; LS = low schizotypy; PBO = placebo; ROI = region of interest; ULO = ulotaront. Error bars are standard errors of the mean.

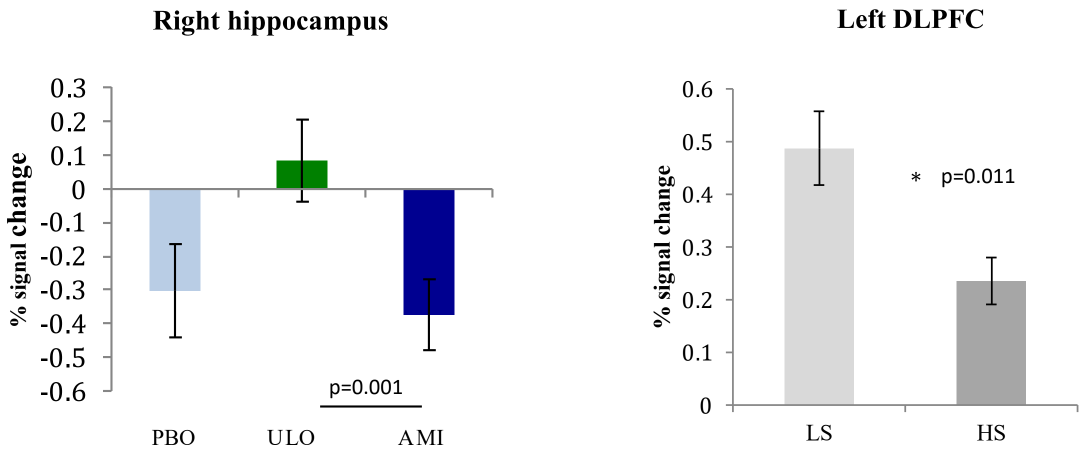

Supplementary Fig. 3. Whole brain analysis N-Back > 0-Back. A) Ulotaront lessened deactivation more than amisulpride in postcentral gyrus (-56 -18 46, voxels 405) and right hippocampus (26 -20 -18, voxels 456). **B)** Effects of schizotypy in frontal pole (20 42 46; voxels, 507) – less deactivation in HS group. **C)** Schizotypy x ulotaront vs placebo interaction in inferior frontal gyrus (-54 -62 -16, voxels 696) and precentral gyrus (26 -16 74, voxels 424). AMI = amisulpride; HS = high schizotypy; LS = low schizotypy; PBO = placebo; ULO, ULOT = ulotaront. Error bars are standard errors of the mean.
*Threshold Z>2.3, * = p<0.05 corrected.*

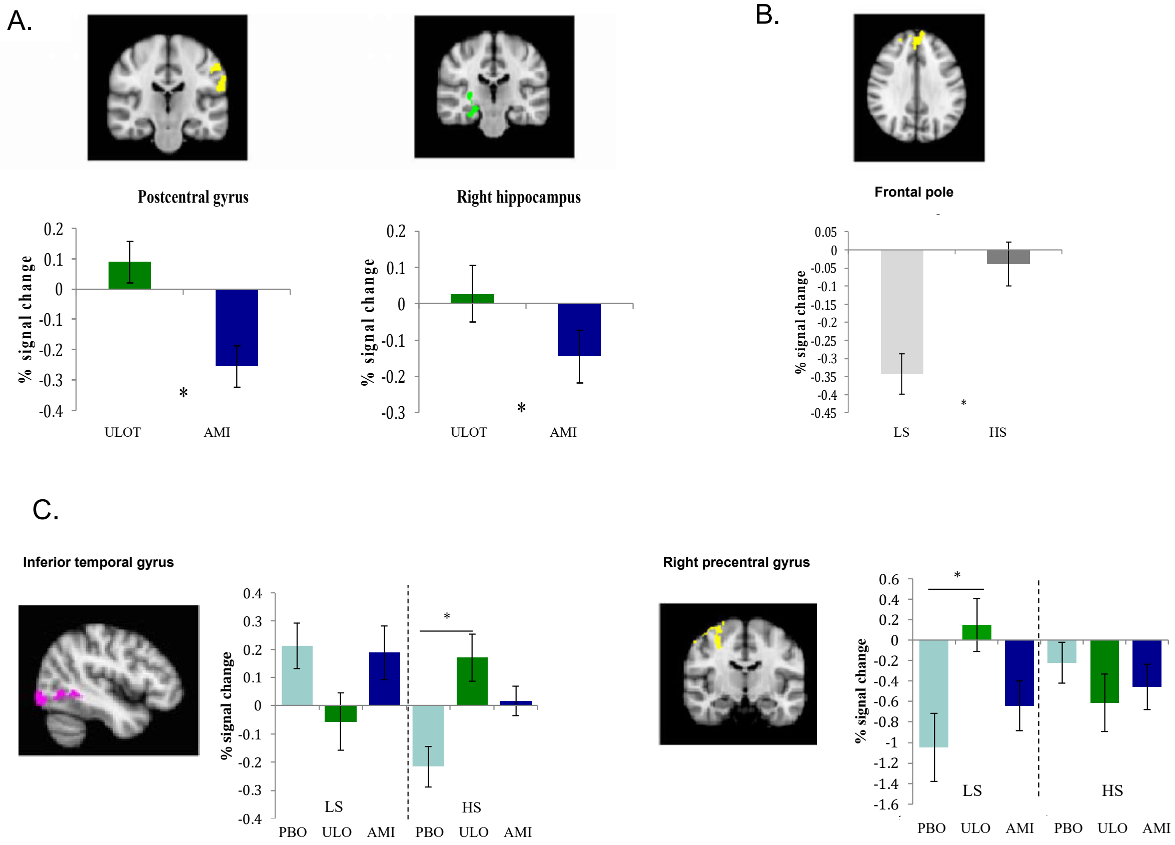

Supplementary Fig. 4. Resting state right executive control network. Significant treatment x schizotypy effects in the placebo vs. ulotaront comparison for inferior frontal gyrus component of right executive control network. HS (light blue bars; LS = dark blue bars) in the placebo group showed significantly reduced connectivity (intensity) and these effects were abolished by ulotaront, but not significantly by amisulpride. HS = high schizotypy; LS = low schizotypy. Error bars are standard errors of the mean.

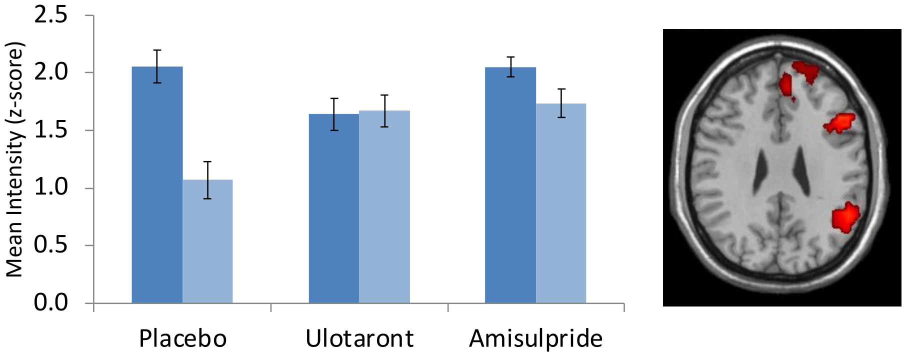

All treatments:	main effect of schizotypy	p=0.001
Placebo vs Ulotaront:	main effect of schizotypy	p=0.011
	treatment*schizotypy	p=0.005
Placebo vs Amisulpride:	*main effect of treatment*	*p=0.063*
	main effect of schizotypy	p=0.001
	*treatment*schizotypy*	*p=0.094*
Ulotaront vs Amisulpride:	main effect of treatment	p=0.038

Supplementary Fig. 5. Arterial spin labelling. Cerebral blood flow (CBF) for placebo versus ulotaront in **A)** thalamus and **B)** DLPFC and ACC. **C)** Effect of treatment versus schizotypy in cingulate gyrus. ACC = anterior cingulate cortex; ASL = Arterial spin labelling; CBF = cerebral blood flow; DLPFC = dorsolateral prefrontal cortex; LS = low schizotypy; HS = high schizotypy; pFWEc = family-wise error–corrected p-value.

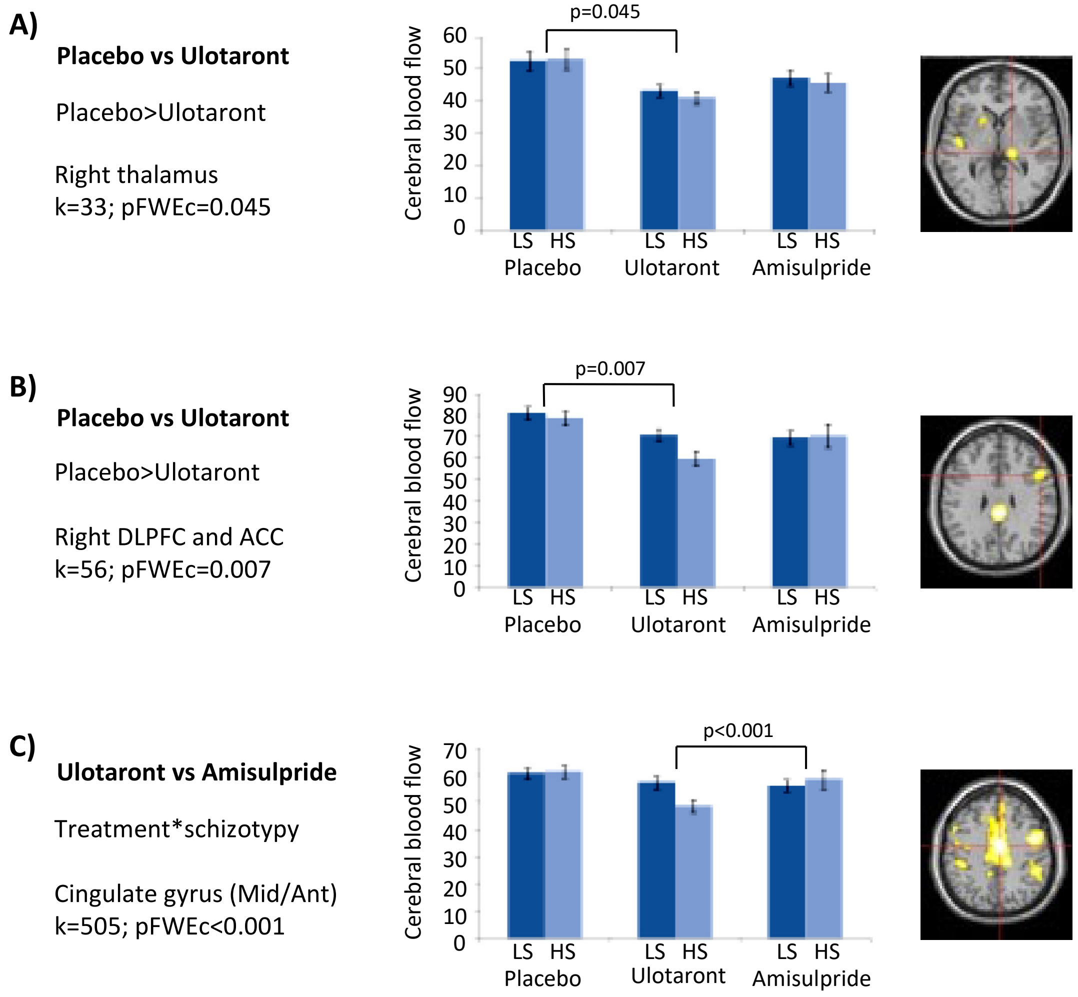

Supplement: Supplementary file 1 — 2022TP001586R Supplementary material [file 41537_2023_385_MOESM1_ESM.pdf]
